# Obesity in Prediabetic Patients: Management of Metabolic Complications and Strategies for Prevention of Overt Diabetes

**DOI:** 10.2174/0118715303282327240507184902

**Published:** 2024-05-20

**Authors:** Marco Chianelli, Marina Armellini, Maria Carpentieri, Carmela Coccaro, Carla Micaela Cuttica, Alessandra Fusco, Simonetta Marucci, Anna Nelva, Maurizio Nizzoli, Maria Chantal Ponziani, Marcello Sciaraffia, Francesco Tassone, Luca Busetto

**Affiliations:** 1 Unit of Endocrinology and Metabolism, Regina Apostolorum Hospital, Albano, Rome, Italy;; 2 Endocrinology and Metabolism Unit, University-Hospital S. Maria della Misericordia, Udine, Italy;; 3 Department of Civil Disability, Istituto Nazionale della Previdenza Sociale, Rome, Italy;; 4 Unit of Endocrinology, Galliera Hospital, Genoa, Italy;; 5 Diabetology Center Villaricca, Azienza Sanitaria 2 Naples, Naples, Italy;; 6 Scienza dell'Alimentazione e Nutrizione Umana, University Campus Biomedico, Rome, Italy;; 7 Unit of Endocrinology and Diabetology, Ospedale degli Infermi, Ponderano, Italy;; 8 Unit of Endocrinology and Metabolism G.B. Morgagni Hospital, Forlì, Italy;; 9 Unit of Diabetes & Metabolic Disease, SS Trinità Hospital, Borgomanero-Arona, Italy;; 10 Unit of Endocrinology, SS. Annunziata Hospital, Taranto, Italy;; 11 Department of Endocrinology, Diabetes & Metabolism, Santa Croce e Carle Hospital, Cuneo, Italy;; 12 Department of Medicine, University of Padova, Padova, Italy

**Keywords:** Obesity, prediabetes, fatty liver disease, diabetes prevention, lifestyle, nutrition, pharmacological therapy, obesity comorbidities

## Abstract

Obesity and prediabetes affect a substantial part of the general population, but are largely underdiagnosed, underestimated, and undertreated. Prediabetes differs from diabetes only in the degree of hyperglycaemia consequent to the progressive decline in residual beta-cell function. Both prediabetes and diabetes occur as a consequence of insulin resistance that starts several years before the clinical onset of overt diabetes. Macrovascular complications in patients with diabetes are mainly caused by insulin resistance. This is why in prediabetes, the overall cardiovascular risk is, by all means, similar to that in patients with diabetes. It is important, therefore, to identify prediabetes and treat patients not only to prevent or delay the onset of diabetes, but to reduce the cardiovascular risk associated with prediabetes. This review provides an overview of the pathophysiology of prediabetes in patients with obesity and the progression toward overt diabetes. We have reviewed nutritional and pharmacological approaches to the management of obesity and reduced glucose tolerance, and the treatment of the major comorbidities in these patients, including hypertension, dyslipidaemia, and Metabolic dysfunction-associated Steatotic Liver Disease (MASLD), has also been reviewed. In patients with obesity and prediabetes, the nutritional approach is similar to that adopted for patients with obesity and diabetes; treatments of dyslipidaemia and hypertension also have the same targets compared to patients with diabetes. MASLD is a critical issue in these patients; in the prediabetic state, MASLD rarely progresses into fibrosis. This highlights the importance of the early recognition of this pathological condition before patients become diabetic when the risk of fibrosis is much higher. It is necessary to raise awareness of the clinical relevance of this pathological condition in order to prompt early intervention before complications occur. The single most important therapeutic goal is weight loss, which must be early and persistent.

## 1 INTRODUCTION

In western countries, the prevalence of obesity is very high; in 2016, in Europe, it was reported at 25.3%, and in the USA, it was 37.3%, showing a steep increase compared to 1975 (10.3% in Europe and 11.7% in the USA) [1]. In people with obesity, alterations in glucose metabolism are very often detected. A recent study showed that in north American young adults aged 19-34, prediabetes was present in 36.9% of people with obesity (compared to 16.6% in normal-weight individuals) [2].

According to the guidelines of the American Diabetes Association (ADA), prediabetes is present if fasting glucose is between 100 and 125 mg/dl (Impaired Fasting Glucose, IFG) or 2 h glucose during a 75 g OGTT is between 140 and 200 mg/dl (Impaired Glucose Tolerance, IGT) or HbA1c is in the range of 5.7-6.4% [3].

Prevalence of prediabetes varies considerably depending on the study population and the parameter chosen; in the general population, it is about 10% in Europe, similar to that of Type 2 Diabetes Mellitus (T2DM) [4]. A recent study, however, reported a 35.6% prevalence of prediabetes in China [5]. In a cohort of 1547 American adults, changing the lower IFG threshold from 110 mg/dL to 100 mg/dL resulted in an increase in prediabetes prevalence from 19.8 to 34.6% [6].

Prediabetes is a relevant risk factor for the development of diabetes [7]. Multiple factors, however, including age, BMI, the presence of gestational diabetes, glycaemic status, and other comorbidities, influence the risk of progression to diabetes [8]. In the Diabetes Prevention Program (DPP), which enrolled high-risk individuals with impaired glucose tolerance, elevated fasting glucose, and elevated BMI, the crude incidence of diabetes within the placebo arm was 11.0 cases per 100 person-years, with a cumulative 3-year incidence of diabetes of 28.9% [9].

In the community-based Atherosclerosis Risk in Communities (ARIC) study, the observational follow-up of older adults (mean age 75 years) with laboratory evidence of prediabetes, but not meeting specific BMI criteria, found the much lower progression to diabetes over 6 years, *i.e*., in 9% of those with A1C-defined prediabetes and 8% of those with impaired fasting glucose [10].

A prospective population-based cohort analysis, however, showed that in individuals aged 45 years, the lifetime risk of progression from prediabetes to diabetes was 74.0% (95% CI 67.6-80.5). The lifetime risks attenuated with advancing age, but increased with increasing BMI and waist circumference [11].

We can conclude, therefore, that in patients with obesity and prediabetes, the rate of progression to diabetes is very high at 45 years of age and decreases if prediabetes occurs later in life, increasing with increasing values of fasting blood glucose/HbA1c and increasing BMI.

Prediabetes, however, is also linked to the development of many pathologies normally associated with T2DM, such as diabetic retinopathy, neuropathy, nephropathy, and above all, macrovascular complications [12, 13]. In addition, there is emerging evidence that prediabetes is associated with cognitive disorders and cancer [13].

A recent study from the UK estimated that half of the study population already had a macro- or microvascular disease at the time of T2DM diagnosis [14]. The study, comparing patients with diabetes to prediabetes, showed the prevalence of co-morbidities as follows: ischaemic heart disease (17.8% *vs*. 20.6%), chronic kidney disease (5.5 *vs*. 3.9%), cerebrovascular disease (5.2 *vs*. 3.5%), peripheral vascular disease (12.9 *vs*. 7.7%%), eye disease (6.5% *vs*. 0.1%), peripheral neuropathy (8.7 *vs*. 2.6%), and autonomic neuropathy (3.6 *vs*. 5.5%) (patients with diabetes *vs*. prediabetes, respectively) [14].

A recent meta-analysis showed that patients with prediabetes, compared to normoglycaemic subjects, had an increased risk of all-cause mortality and cardiovascular disease [7]. In the general population, impaired glucose tolerance carried a higher risk of all-cause mortality, coronary heart disease, and stroke than impaired fasting glucose. The risk of all-cause mortality associated with impaired fasting glucose was mainly attributed to fasting plasma glucose concentrations in the range of 110-125 mg/dl. The risk of cardiovascular events and coronary heart disease was increased in the general population with a HbA1c concentration of 5.7-6.4%. It was also found that prediabetes was associated with an increased risk of mortality and cardiovascular outcomes in patients with established atherosclerotic cardiovascular disease [7].

Prediabetes may, however, be reversible through the implementation of lifestyle modification programs based upon the adoption of a healthier diet and increased levels of physical activity and, in selected cases, medical treatment [15].

A commonly cited statistic states that 1:3 Americans have prediabetes, and that 90% of patients with prediabetes are not aware of their condition [4]. The identification of obese subjects with prediabetes, therefore, is extremely important; a percentage of these patients develops cardiovascular disease and other diabetes- and obesity-related complications. Early treatment of metabolic impairment and obesity in these patients can lead eventually to the prevention of overt diabetes and metabolic complications. Probably the most important single action in these patients is the treatment of obesity [16].

In this review, we have looked into the mechanisms of development of diabetes, the strategies for preventing diabetes, treating obesity, and its metabolic consequences in this group of patients.

## 2 HOW PATIENTS WITH OBESITY AND PREDIABETES PROGRESS TO OVERT DIABETES

Is obesity the cause of diabetes? Several data seem to point to a direct relationship between obesity and the development of diabetes.

We know that childhood obesity is associated with an increased risk of developing diabetes in adult age. A recent study on patients with obesity and without diabetes, with respect to a control group of lean subjects, described, in the absence of obesity, a reduction of incidence of diabetes of about 60% in 10 years.

Results of a study have also demonstrated that the presence of obesity, 1^st^ to 2^nd^ degree, causes an increase of 4 times in the onset of diabetes [17]. On the contrary, the reduction of weight of just 4 BMI points, from obesity to overweight, halves the likelihood of developing diabetes [17].

In summary, 87.5% of people with diabetes are obese or overweight and 13.8% of patients with obesity have diabetes. However, 86.2% of patients with obesity do not develop type 2 diabetes [2, 18]. Why do some patients with obesity develop diabetes and others do not? To clarify this aspect, it is necessary to investigate the mechanisms at play in patients with obesity with or without diabetes.

### 2.1 Insulin Resistance in Patients with Obesity and without Diabetes

86.2% of patients with overweight or obesity have insulin resistance, but they do not develop diabetes. Insulin resistance, as measured by the Homeostasis Model of Assessment of Insulin Resistance (HOMA-IR), in patients with obesity not predisposed to diabetes, is not genetically determined. It is a consequence of weight gain as fat storage increases, adipocyte hypertrophy becomes less responsive to insulin, which may also be due to oxidative stress that leads to the inactivation of the glucose transporter GLUT4 [19]. Insulin resistance of the adipose tissue, therefore, is different from insulin resistance present in patients with diabetes and prediabetes, typically muscular in origin. Adipose insulin resistance alone is unable to determine the onset of the disease.

Insulin resistance in patients with obesity and without diabetes (BMI 46.5, HOMA-IR 5.8 ± 1.9) is significantly lower compared to patients with obesity and diabetes (BMI 41.8, HOMA 10.3 ± 7.7), as demonstrated in recent studies [2].

Finally, an increase in insulin and glucagon has been described in patients with obesity and diabetes or reduced tolerance to carbohydrates, while in patients with obesity with normal glucose tolerance, normal glucagon values are recorded reflecting different pathogenetic pathways in the two conditions [20].

### 2.2 Insulin Resistance in Patients with Obesity and Prediabetes or Diabetes

In normal subjects, muscles take up 80% of circulating glucose, up to a maximum of 10 mg/kg/min 60 minutes after a meal. In patients with obesity predisposed to diabetes, insulin resistance of the muscle is genetically determined and causes a reduction in muscle uptake of glucose up to 50%, with a consequent intracellular deficit of the energy substrates, glucose and fatty acids, which is associated with an increase in glycaemic values [21]. The increase in blood sugar causes a compensatory increase in insulin production, which determines a new balance capable of ensuring adequate intracellular levels of fatty acids and glucose to support normal cardiac and muscle activity.

#### 2.2.1 Adipose Tissue, Inflammation, and Insulin Resistance

**Fig. (1):**
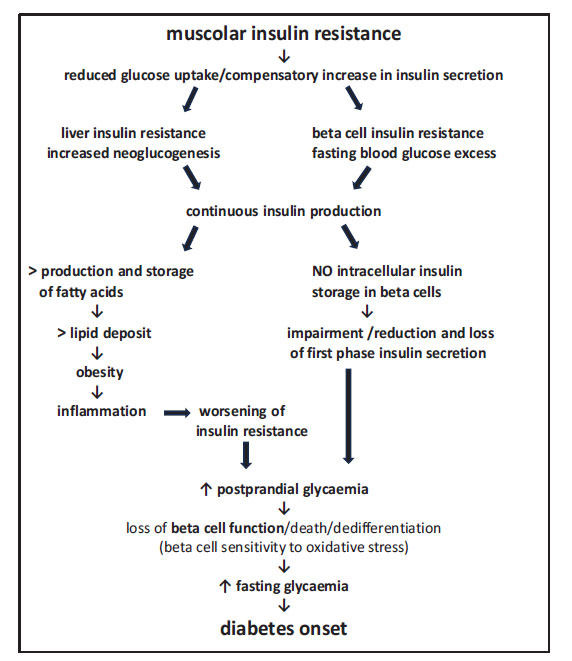
Simplified scheme of progression towards diabetes in genetically predisposed subjects. Increased glucose availability consequent to reduced muscular uptake induces compensatory increase in insulin production and promote reduction of liver and beta cell insulin sensitivity; this further increases glucose levels and cause development of obesity, inflammation and post-prandial hyperglycaemia; excess insulin production, increased levels of glucose and of fatty acid determine, in predisposed individuals, oxidative stress of beta cells with loss of function and onset of diabetes.

Excess glucose is transported in an insulin-dependent manner within the adipocyte to be transformed into fatty acids and glycerol 3-phosphate and form triglycerides with consequent expansion of adipose tissue and worsening of obesity. Obesity, in turn, causes low-grade Th1-type systemic inflammation, characterized by increased production of cytokines, which, directly or indirectly, block the action of insulin, worsening peripheral insulin resistance. The adipose tissue also expands in the liver and the islets of Langerhans, causing an increase in tissue macrophages, in turn leading to inflammatory and oxidative stress (Fig. **1**) [22]. The following aspects also occur:

- Lack of insulin has a suppressive effect on hepatic gluconeogenesis;
- Reduced response of beta cells to glucose leads to a progressive reduction in insulin production;
- Lack of suppressive effect on the alpha cells affects the secretion of glucagon following a meal.

#### 2.2.2 The Reduction of Insulin Production as a Key to the Development of Diabetes

In normoglycaemic patients without insulin resistance, insulin is produced only following a meal that causes blood sugar to rise. In patients with obesity with a predisposition to diabetes, on the other hand, insulin resistance causes continuous hepatic production of glucose and reduced muscle uptake with excess glucose in the blood even when fasting. To maintain normal blood glucose levels, insulin is produced continuously, even during fasting. Over time, there is a reduction of intracellular deposits of insulin in the beta cells and a progressive reduction of the first phase of insulin response; thus, post-prandial glycaemia begins to increase (Fig. **1**) as does metabolic imbalance, leading to an increase in lipid concentrations.

Chronic hyperlipemia causes an increase in Reactive Oxygen Species (ROS) to which beta cells show greater susceptibility than other cells. Oxidative stress contributes to the reduction of the beta cells’ ability to produce insulin and can induce cell death *via* apoptosis. Finally, the prolonged increase in insulin synthesis damages the endoplasmic reticulum due to the pathological intracytoplasmic accumulation of proteins [23, 24].

A further increase in blood glucose is then observed, which further reduces the secretory capacity of beta cells (glucotoxicity), inducing the release of chemokines that mediate the metabolic, inflammatory, and oxidative damage to the beta cell.

#### 2.2.3 Genetic Predisposition to Loss of Beta Cell Function

In patients with obesity and insulin resistance not predisposed to diabetes, beta cells, by means of a compensatory increase in number and volume, are able to guarantee the production of high quantities of insulin for decades without showing functional exhaustion, while maintaining normal glycaemic values. In these individuals, beta cells seem more resistant to inflammation and oxidative stress as a result of the presence of normal levels of enzymes that inactivate oxygen free radicals and, probably, also due to the lower metabolic stress resulting from the increase in beta cell mass [24].

Clinical and experimental evidence indicates that genetically determined abnormalities play a major role in the pathogenesis of type 2 diabetes mellitus. Many genetic defects identified in genome-wide association studies are involved in beta cell function [25, 26]. There is extensive data on these with more than 600 genes now associated with diabetes, but most of them being in non-coding regions [27]. A recent study, using isogenic human embryogenic stem cell knockout for test genes, investigated the role of genes that have been recognised as susceptibility genes for type 2 diabetes, including B cell generation, differentiation efficiency, insulin production and secretion, and B cell survival after lipotoxic exposure. Results showed at least four susceptibility genes to be associated with insulin production and two with B cell survival, and also revealed pathways involved in insulin production and apoptosis [28].

In fact, in patients with obesity and prediabetes, beta cells are genetically susceptible to functional and oxidative damage associated with insulin resistance and, after years of metabolic stress due to increased insulin production, show a progressive reduction, up to functional exhaustion, through three main mechanisms, including functional metabolic alterations, cell death, and loss of differentiation, an effect considered to be the most relevant to date.

The combined effect of these factors, in the presence of a genetic predisposition, ultimately leads to the onset of type 2 diabetes mellitus, the final result of a complex interaction between environmental factors and genetic factors [24].

### 2.3 Final Remarks on the Progression to Overt Diabetes

The development of diabetes in people with obesity and prediabetes is a complex phenomenon, not completely understood, that results from the complex interplay between environment and genetics. In the first instance, predisposed individuals become obese as a result of the exposition to an obesogenic environment. The onset of insulin resistance of the muscle and the adipose tissue and then of the liver and beta cells, in the presence of elevated levels of circulating glucose and fatty acids, exposes the beta cell to glucose- and lipotoxicity that, in genetically predisposed individuals, results in beta cell death and/or dedifferentiation. The genetic predisposition of beta cells to oxidative stress induced by lipotoxicity and glucose toxicity is central to the decrease of insulin secretion first and the development of diabetes.

## 3 LIFESTYLE AND DIET

In recent years, a considerable number of studies have been published analysing the beneficial effects of diet on the incidence of diabetes. The most recent reviews and meta-analyses compare low-fat or low-carbohydrate diets, Mediterranean-type diets, and vegetarian or vegan diets, all of which are considered healthy dietary patterns *versus* Western or canteen-based diets. As a novelty, recent studies have also evaluated other types of interventions, such as mobile applications and kitchen laboratories [29].

The indications provided by American and European Guidelines [15, 30] indicate that to reduce the risk of DM and cardiovascular events, it is necessary to:

- Reduce calorie intake to lower excessive body weight in patients with DM.
- Consume a Mediterranean diet supplemented with olive oil and/or nuts, which reduces the incidence of major CV events.
- Practice moderate-to-vigorous physical activity of ≥150 min/week, which is recommended for the prevention and control of DM.

Part of these indications derives from the results of the DPP, DPPOS (Diabetes Prevention Program Outcomes Study), and other studies, such as Da Qing Diabetes Prevention Study and the Finnish Diabetes Prevention Study (DPS), which will be dealt with in detail in a later chapter [9, 31, 32].

Physical activity is a fundamental factor in weight control and there are numerous studies that have evaluated its effectiveness not only for the purpose of weight reduction, but also for the prevention of cardiovascular and metabolic complications. Physical activity is an essential part of any weight loss program not only because it increases energy expenditure and reduces insulin resistance, but it is a key factor for the prevention of metabolic adaptation typical of patients with obesity [33]. In patients with obesity, after losing weight with a diet without supplementation with physical activity, a reduction in resting energy expenditure is noted that is far greater compared to that expected for the amount of weight loss; metabolic adaptation can be prevented if structured physical activity is included in the weight-loss program [33]. Metabolic adaptation is an important limiting factor for weight loss and predisposes to weight regain. Resistance activity, furthermore, should also be included to minimise loss of lean mass. Metabolic adaptation is an important limiting factor for weight loss and predisposes to weight regain. Resistance activity, furthermore, should also be included to minimise loss of lean mass.

Physical activity alone, however, in the presence of obesity reduces cardiovascular complications, but these remain more serious in people with obesity compared to normal-weight individuals of the same age, showing that weight control is essential for the control of comorbidities [34, 35].

### 3.1 Nutrition, Glucose Metabolism, Obesity, and Prediabetes

In a recent consensus by the American Diabetes Association on nutrition therapy of adults with diabetes or prediabetes was clearly stated that patients with prediabetes must have an individualized approach based on cultural background, BMI, age, and other parameters, with the aim of preventing the development of diabetes by weight control and improved glucose metabolism [36]. Particularly important is weight management, which is crucial for diabetes prevention. When the prediabetic state is diagnosed in people with obesity, the nutrition approach is to promote sustained weight loss of at least 7% [37] by fat and calory restriction. Once weight control is achieved, diet is aimed particularly at controlling glucose metabolism and other risk factors, such as blood pressure and lipids. The principles of nutrition therapy described for diabetes can be applied to the nutritional management of people with prediabetes.

However, one diet “does not fit all” and different approaches can be used. The main dietary approaches alongside nutrition principles have been described.

Low carbohydrate diets, Dietary Approaches to Stop Hypertension (DASH), vegetarian diets, and Mediterranean diets are some of the dietary models recommended by the American and European guidelines for the management of type 2 diabetes. The Mediterranean diet has been the most studied and is linked to greater glycaemic control than other dietary patterns. Adherence to this dietary pattern has been found to be associated with lower body weight, and it has been shown to prevent long-term weight regain and to induce significant weight loss, with or without energy restriction [29, 38].

There are numerous and long-lasting studies supporting the Mediterranean diet, such as PREDIMED or DASH, which show an average reduction of approximately 20% for the onset of diabetes and approximately 52% for cardiovascular risk. There is a considerable number of publications on the Mediterranean diet and its specific components, some of which have been individually reported to reduce the incidence of diabetes. Extensive data on the vegetarian diet show a reduction in the risk of developing DM2; studies show that the effectiveness of the vegetable diet is also strongly influenced by the type of vegetable oils used [29].

The Mediterranean diet is able to reduce central obesity and, in turn, reduce chronic diseases related to obesity, such as T2D. In fact, this diet is superior to low-fat ones for long-term weight loss. Additionally, it is associated with a more significant improvement in insulin resistance in people with obesity compared to other low-energy dietary approaches.

The effects exerted by the Mediterranean diet on T2D development could probably be attributed to its anti-inflammatory/antioxidant compounds. It is known that individuals with diabetes have a significantly lower level of ascorbic acid, β-carotene, and α-tocopherol/cholesterol ratio than non-diabetic ones. For example, the Mono-unsaturated Fatty Acids (MUFA), the main fat of EVOO (especially oleic acid), are thought to counteract the effect promoted by Saturated Fatty Acids (SFA), which decrease the Insulin Sensitivity (IS) of peripheral tissues. Quercetin can enhance the uptake of glucose in skeletal myocytes through an AMPK-dependent up-regulation of glucose transporter GLUT-4; PUFA (Poly-unsaturated Fatty Acids) can stimulate G-protein-coupled receptors, such as GPR120, leading to increased secretion of GLP1 from enteroendocrine L-cells and reducing the level of interleukin-6 and C-reactive protein [29, 39].

Significant evidence shows that the Mediterranean diet is able to positively modify the gut microbiota, which we know to be one of the factors related to the development of TD2. The Mediterranean diet can stimulate the production of Short-chain Fatty Acids (SCFA) in the gut microbiota, whose values are reduced in TD2 due to the elevated content of dietary fibres. Moreover, new studies seem to show that physical activity too can positively influence the intestinal gut [40, 41].

### 3.2 Nutritional Aspect

The National Academy of Medicine recommends individualized Medical Nutrition Therapy (MNT), provided by a Registered Dietitian Nutritionist (RDN) upon physician referral, as part of the multidisciplinary approach to diabetes care with the goals of improving eating habits, increasing moderate-intensity physical activity to at least 150 min per week, and achieving and maintaining 7-10% loss of initial body weight if needed [30, 23]. More intensive intervention programs are the most effective in decreasing diabetes incidence and improving Cardiovascular Disease (CVD) risk factors [29, 30, 42, 43].

#### 3.2.1 Macronutrients

Although numerous studies have attempted to identify the optimal mix of macronutrients for the eating plans of people with diabetes, a systematic review [44] has found no ideal or broadly applicable mix, and that macronutrient proportions should be individualized for each patient. It has been observed that people with diabetes, on average, consume about the same proportions of macronutrients as the general public: 45% of their calories from carbohydrates; 36-40% of calories from fat, and the remainder (16-18%) from protein. Regardless of the macronutrient mix, total energy intake should be appropriate to attain weight management goals. Furthermore, individualization of the macronutrient composition depends on the status of the individual, including their metabolic goals (glycaemia, lipid profile, *etc*.), physical activity, food preferences, and availability.

#### 3.2.2 Carbohydrate

Carbohydrate is a readily used source of energy, having a primary dietary influence on postprandial blood glucose. Foods containing carbohydrates with various proportions of sugars, starches, and fibre have a wide range of effects on the glycaemic response. The quality of carbohydrate foods selected, which should be ideally rich in dietary fibre, vitamins, and minerals, and low in added sugars, fats, and sodium, should be addressed as part of an individualized eating plan that includes all components necessary for optimal nutrition [45].

#### 3.2.3 Fibre

People with diabetes should consume at least the amount of fibre recommended by guidelines, with at least half of grain consumption being whole intact grains. Other sources of dietary fibre include non-starchy vegetables, avocados, fruits, and berries, as well as pulses, such as beans, peas, and lentils. Meeting the recommended fibre intake through foods that are naturally high in dietary fibre, as compared to supplementation, is encouraged for the additional benefits of coexisting micronutrients and phytochemicals [45].

#### 3.2.4 Protein

There is limited research on people with diabetes or prediabetes without kidney disease with respect to the impact of various amounts of protein consumed. Some comparisons of protein amounts have not demonstrated differences in diabetes-related outcomes [46].

#### 3.2.5 Fat

The National Academy of Medicine has defined an acceptable macronutrient distribution for total fat for all adults to be 20-35% of total calorie intake [47]. Eating patterns that replace certain carbohydrate foods with those higher in total fat, however, have demonstrated greater improvements in glycaemia and certain CVD risk factors (serum HDL Cholesterol (HDL-C) and triglycerides) compared to lower fat diets. The types or quality of fats in the eating plans may influence CVD outcomes beyond the total amount of fat. Large epidemiologic studies have found that consumption of polyunsaturated fat or biomarkers of polyunsaturated fatty acids is associated with a lower risk of type 2 diabetes. The intervention in the PREvencion con DIeta MEDiterranea (PREDIMED) study, comparing a Mediterranean-style eating pattern supplemented either with extra-virgin olive oil or with nuts *versus* a control diet, reduced incidence of type 2 diabetes among people with prediabetes at high cardiovascular risk at baseline.

### 3.3 Eating Patterns

The most robust research available on eating patterns for the prevention of prediabetes or type 2 diabetes is on Mediterranean-style, low-fat, or low-carbohydrate diets [48]. The PREDIMED trial, a large RCT, compared a Mediterranean-style to a low-fat diet for the prevention of the onset of type 2 diabetes, with the Mediterranean-style diet resulting in a 30% lower relative risk. Epidemiologic studies correlate Mediterranean-style [49], vegetarian [50, 51], and Dietary Approaches to Stop Hypertension (DASH) [52] diets with a lower risk of developing type 2 diabetes, indicating no effect for low carbohydrate dietary patterns. Several large type 2 diabetes prevention RCTs used low-fat dietary plans to achieve weight loss and improve glucose tolerance, and some demonstrated a decreased incidence of diabetes. Given the limited evidence, it is unclear which of the dietary patterns are optimal.

A variety of dietary patterns (combinations of different foods or food groups) is acceptable for the management of diabetes.

Until the evidence surrounding the comparative benefits of different dietary patterns for specific individuals strengthens, healthcare providers should focus on the following key factors that are common among the patterns:

- Emphasize non-starchy vegetables.
- Minimize added sugars and refined grains.
- Choose whole foods over highly processed foods to the extent possible.

Reducing overall carbohydrate intake for individuals with diabetes has demonstrated the strongest evidence for improving glycaemia and may be applied to a variety of dietary patterns that meet individual needs and preferences.

For certain adults with type 2 diabetes who do not meet the glycaemic targets or in cases where reducing anti-glycaemic medications is a priority, reducing overall carbohydrate intake with low or very low carbohydrate dietary plans is a viable approach.

The traditional Mediterranean diet is characterized by cooking seasonal and local products and enjoying socialization with meals. It consists of a daily abundance of vegetables, a variety of minimally processed whole grain bread and other cereals and legumes as the staple food, nuts and seeds, and fresh fruit as the typical daily dessert; sweets based on nuts, olive oil, and honey are consumed only during celebratory occasions. In this diet, cold-pressed extra-virgin olive oil, nuts, and seeds are the principal sources of fat, and a low to moderate quantity of dairy products (mainly local cheese and yogurt) is consumed. It also involves moderate consumption of fish, poultry, and eggs, a low consumption of red meat (once a week approximately), and a moderate consumption of wine, normally with meals [45].

### 3.4 Energy Balance and Weight Management

To support weight loss and improve HbA1c levels, CVD risk factors, and quality of life in overweight adults or adults with obesity and prediabetes or diabetes, MNT and DSMES services should include an individualized dietary plan in a format that results in an energy deficit in combination with enhanced physical activity.

For adults with type 2 diabetes who are not taking insulin and who have limited health literacy or numeracy, or who are older and prone to hypoglycaemia, a simple and effective approach to glycaemia and weight management emphasizing appropriate portion sizes and healthy eating may be considered.

In type 2 diabetes, 5% weight loss is recommended to achieve clinical benefit, and the benefits are progressive. The goal for optimal outcomes is 15% or more when needed and can be feasibly and safely accomplished. In prediabetes, the goal is 7-10% for preventing progression to type 2 diabetes. In certain individuals with type 2 diabetes, an overall healthy dietary plan that results in an energy deficit in conjunction with weight loss medications and/or metabolic surgery should be considered to help achieve weight loss and maintenance goals, lower HbA1c, and reduce CVD risk. In conjunction with lifestyle therapy, medication-assisted weight loss can be considered for people at risk for type 2 diabetes when needed to achieve and sustain 7-10% weight loss.

People with prediabetes and of a healthy weight should be considered for lifestyle intervention involving both aerobic and resistance exercise and a healthy eating plan, such as a Mediterranean-style dietary plan [36].

### 3.5 Sleep, Glucose Metabolism, Obesity, and Prediabetes

Sleep quality and duration are an important part of the lifestyle that is normally underestimated and under-recognised.

Sleep deprivation can interfere with energy balance and cause weight gain through three distinct pathways: increased hunger, greater time to eat, and/or a decrease in energy expenditure. Significant weight gain may lead to insulin resistance, which can further encourage obesity. Furthermore, up to 20% of overweight and obese people suffer from Sleep-disordered Breathing (SDB), which is an independent risk factor for insulin resistance.

Indeed, a considerable body of literature suggests that sleep fragmentation, hypoxia, and low levels of deep non-rapid Eye Movement (REM) sleep, all of which occur in SDB, may all contribute to decreased insulin sensitivity.

The orexin system is expected to play an important role in the interactions between sleep and food. Orexins A and B are two different peptides produced mainly by neurons in the lateral hypothalamus. These neurons serve an important function in maintaining awareness and increasing feeding, especially when typical food intake is low. Feeding necessitates the maintenance of alertness and the orexin system. Orexin neurons are active during waking hours, in sync with neuronal activity in other arousal centres, and inactive during slow-wave sleep.

Consistent with the fact that sympathetic nervous activity is higher during waking than during sleeping, orexin activity is associated with increased sympathetic tone, and orexin peptides have been shown to have excitatory effects on neurons in the nucleus tractus solitarius and the hypothalamic paraventricular nucleus. In animal studies, sleep deprivation increases central orexin activity. Higher orexinergic activity increases sympathetic nerve tone and the activity of arcuate nucleus Neuropeptide Y (NPY) neurons, which stimulate appetite.

According to studies, decreasing sleep duration may raise the risk of weight gain and obesity by upregulating hunger also through a drop in leptin, an increase in ghrelin, and a decrease in insulin sensitivity. Limited evidence from population research suggests that leptin plays a role in the connection between short sleep and increased BMI. Leptin and ghrelin are hypothesized to work in tandem as opposing metabolic counterparts for body mass homeostasis.

Sleep deprivation's increased sympathetic nervous activity may contribute to peripheral effects, such as decreased leptin release from adipocytes, decreased total body insulin sensitivity, and insufficient compensation by β-cells [53]. The increased ghrelin/leptin ratio is an independent risk factor for the development of obesity and T2DM.

Slow Wave Sleep (SWS), considered the most restorative sleep stage, has been linked to lower heart rate, blood pressure, sympathetic nerve activity, and cerebral glucose utilization as compared to the awakening stage. SWS causes the production of anabolic growth hormone while inhibiting the stress hormone cortisol.

Furthermore, SWS has significant modulatory effects on endocrine release. The Hypothalamic-Pituitary-Adrenocortical (HPA) system's hormones are repressed, while Growth Hormone (GH) and prolactin levels are raised. Both GH and cortisol play key roles in glucose metabolism. According to laboratory research, acute complete sleep deprivation has a negative impact on the levels of several metabolic hormones [54].

Studies on normal sleepers have demonstrated that nocturnal GH release is reduced in people who are completely sleep-deprived, but it then increases during daytime recovery sleep (with the opposite occurring for cortisol release).

In patients with obesity, a phenomenon known as 'leptin resistance' has been suggested to explain the contradictory finding that most persons with obesity have high, rather than low, plasma leptin levels, despite their plentiful energy stores.

It appears that leptin binds to circulating C-reactive Protein (CRP), an inflammatory marker that is increased in obesity. Higher CRP levels could lead to a decrease in unbound, free leptin, resulting in a reduction in its central effects.

Several investigations of acute total sleep deprivation and recurrent partial sleep deprivation in healthy lean people have found that CRP levels rise with sleep loss. The combination of lower leptin levels and higher CRP concentrations in sleep-deprived individuals may magnify the effects of leptin reduction alone [53].

**Fig. (2):**
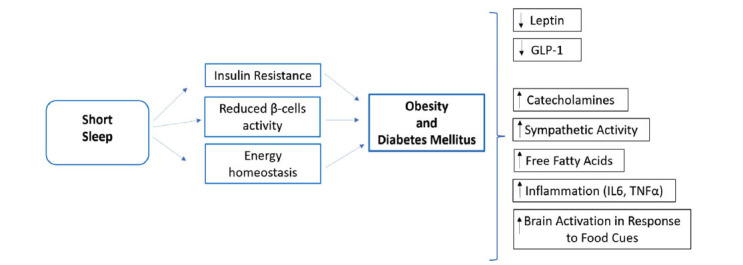
Correlations between sleep quality and development of obesity/type 2 diabetes mellitus.

Sleep quality and duration are an important part of a lifestyle that is normally underestimated and under-recognised. In a large meta-analysis, habitual short sleep duration and experimental sleep restriction were associated with sympathetic predominance either for reduced vagal tone or increased sympathetic activity; this can lower the response of β-cells to glucose and reduce insulin sensitivity without a compensatory increase in insulin secretion, suggesting reduced β-cell function in people without T2DM, especially in men compared to women (Fig. **2**).

Short sleep duration is also associated with increased ghrelin/leptin ratio and is an independent risk factor for the development of obesity and T2DM. Early evidence suggests that sleep manipulation has a favourable impact on weight loss or body composition as a part of lifestyle intervention (calorie restriction) [55].

Prediabetes is positively associated with poor sleep. Rafalson and colleagues, in 2010, tested the hypothesis of short sleep duration at baseline as associated with an increased likelihood of developing Impaired Fasting Glucose (IFG; blood glucose levels between 100 and 125 mg/dl) independent of diabetes risk factors and several confounding variables, in 971 persons with normoglycemia (blood glucose levels below 100 mg/dl) at the baseline. The development of IFG was followed up. After six years, 91 persons developed IFG and were compared to 272 persons who remained normoglycaemic at follow-up. Persons with short sleep duration (less than six hours) showed a significant, three-fold increased likelihood of developing IFG. This association was explained in part by insulin resistance. Persons who slept more than 8 hours did not show an increased risk of developing IFG [56, 57].

Finally, a systematic review and meta-analysis on the effects of sleep behaviours in patients with prediabetes concluded that sleeping less than 6 hrs is associated with an increased risk of progression to type 2 diabetes [58]. In conclusion, identifying specific sleep behaviours might serve as a potential target to aid current measures for the prevention of prediabetes and its progression to T2DM.

### 3.6 Studies on Lifestyle Intervention

Many studies have evaluated lifestyle efficacy in preventing diabetes in people with prediabetes. The Da Qing Diabetes Prevention Outcome Study recruited 577 subjects with IGT who were assigned to three kinds of intervention groups (diet, exercise, or diet plus exercise) or the control group. The aim of the study was to identify subjects who developed type 2 diabetes during the 6-year follow-up period. The cumulative incidence of diabetes at 6 years was 67.7% in the control group, 43.8% in the diet group, 41.1% in the exercise group, and 46.0% in the diet-plus-exercise group, respectively (*p* < 0.05). Each of the active intervention groups differed significantly from the control group (*p* < 0.05). In an analysis adjusted for differences in baseline BMI and fasting glucose, the diet, exercise, and diet-plus-exercise interventions were associated with 31% (*p* < 0.03), 46% (*p* < 0.0005), and 42% (*p* < 0.005) reductions in risk of developing diabetes, respectively [59].

The Diabetes Prevention Program randomly assigned 3234 nondiabetic persons with IFG (Impaired Fasting Glucose) or IGT (Impaired Glucose Tolerance) to placebo, metformin (850 mg twice daily), or a lifestyle modification program with the aim of obtaining at least a 7% weight loss and at least 150 minutes of physical activity per week. During the 3-year follow-up period, the lifestyle interventions reduced the incidence of diabetes by 58% and the treatment with metformin by 31% as compared to the placebo. The difference between lifestyle intervention and metformin was statistically significant [9]. In the 10-year follow-up of the Diabetes Prevention Program, diabetes incidence was reduced by 34% in the lifestyle group and 18% in the metformin group compared to placebo, showing that prevention or delay of diabetes with lifestyle intervention or metformin can persist for at least 10 years [60].

The Finnish Diabetes Prevention Program randomly assigned 522 middle-aged, overweight subjects with impaired glucose tolerance to either the intervention group or the control group. Each subject in the intervention group received structured information with the aim of reducing weight and total intake of fat, including saturated fat, and increasing intake of fiber and physical activity. An oral glucose-tolerance test was performed annually; the diagnosis of diabetes was confirmed by a second test. The mean duration of follow-up was 3.2 years. The cumulative incidence of diabetes after four years was 11% in the intervention group and 23% in the control group. During the trial, the risk of diabetes was reduced by 58% (*p* < 0.001) in the intervention group [32]. In the extended follow-up of the Finnish Diabetes Prevention Study, the authors continued to follow the persons who originally achieved lifestyle changes and observed that risk reduction remained after discontinuation of active counselling (36% reduction in relative risk). During the total follow-up, the incidence of type 2 diabetes was 4.3 and 7.4 per 100 person-years in the intervention and control groups, respectively. Risk reduction was related to success in achieving the goals of weight loss, changes in eating habits, and increased physical activity [61].

The authors of the Diabetes Prevention Program (DPP) explored the contribution of changes in weight, diet, and physical activity to the risk of developing diabetes among persons randomly assigned to lifestyle intervention. Weight loss was the dominant predictor of reduced diabetes incidence (hazard ratio per 5-kg weight loss 0.42; *p* < 0.0001). For every kilogram of weight loss, there was a 16% reduction in risk, adjusted for changes in diet and activity. Among 495 participants not meeting the weight loss goal at year 1, those who achieved the physical activity goal had 44% lower diabetes incidence. The metabolic syndrome affected approximately half of the participants in the Diabetes Prevention Program at baseline. In life-table analyses (log-rank test), incidence of the metabolic syndrome was reduced by 41% in the lifestyle group (*p* < 0.001) and by 17% in the metformin group (*p* = 0.03) compared to placebo. Three-year cumulative incidences were 51%, 45%, and 34% in the placebo, metformin, and lifestyle groups, respectively [62].

## 4 VERY LOW CALORIE KETOGENIC DIET (VLCKD) AND PREDIABETES

Recent meta-analyses have demonstrated the beneficial effects of a Very Low Calorie Ketogenic Diet (VLCKD) on weight loss in patients with obesity [63, 64].

The ketogenic diet is a reversal of the current food pyramid supported by the dietary guidelines. Thus, instead of a diet rich in carbohydrates, it is high in fat. The resulting carbohydrate restriction lowers blood glucose levels, and the following decrease in insulin levels causes the body to change from a condition of storing fat to a state of fat oxidation. Once Free Fatty Acids (FFA) are utilized as a primary fuel source in the liver, the production of ketone bodies is started, a process known as ketogenesis. During ketosis, three major ketone bodies are synthetised and used by the body as energy sources: acetone, acetoacetate, and β-hydroxybutyrate. All cells that contain mitochondria can meet their energy demands with ketone bodies, including brain and muscle. In addition, β-hydroxybutyrate acts as a signal molecule in the brain, playing a role in suppressing appetite. Several studies suggest that a protocolled VLCKD is more effective in favouring weight loss as compared to a standard LCD.

In a recent and exhaustive review, results of studies on VLCKDs have been presented and discussed, with a particular reference to the protocolled VLCKD [65]. It consists of a multiphase diet organized in an active phase, a metabolic stabilization phase, and a maintenance phase. First, patients start with a short period of VLCKD, which provides daily calories below 700-800 kcal/day and an amount of protein equivalent to 0.8-1.2 g/day per kg of ideal body weight, mainly as replacement meals. It is noteworthy that, given the known deficiencies of restricted-calorie diets, adequate micronutrient supplementation should always be warranted during this phase [66, 67]. The first step is maintained until the 80% reduction of the excess body weight is met, usually lasting up to 12 weeks. Then, a gradual reintroduction of natural protein foods is performed, still keeping the overall calories below 700-800 kcal/day. In the second and third phases, calories and carbohydrates are gradually raised to a Low-calorie Diet (LCD), and then to a balanced diet with a daily intake of 800-1500 and 1500-2250 kcal, respectively.

Concerning the body composition in protocolled VLCKD, a recently published study addressed this issue [62]. Twenty patients with obesity were included, and body composition was assessed by three standardized methods: 1) Dual-energy Xray Absorptiometry (DXA), 2) air displacement plethysmography (Bod-Pod) techniques, and 3) Multifrequency Bioelectrical Impedance (MF-BIA). DXA represents the gold standard for the evaluation of body composition [68, 69]. Changes in fat-free mass were limited to -4 kg, and this can be considered an acceptable result in front of the observed much higher reduction in fat mass. The protocolled VLCKD was able to significantly reduce body weight at the expense of total fat mass and visceral fat mass, with no or minor effects on bone mass and muscular mass and function [70].

VLCKD use has also been linked to the recovery of the first phase of insulin secretion and, consequently, to a significant reduction in the need for glucose-lowering agents, including insulin [67].

Recently, the American Diabetes Association (ADA) has included the use of VLCKD as a viable therapeutic option for the treatment of T2DM and of patients with prediabetes and obesity [36].

In patients with obesity, the effect of VLCKD is powerful in reducing plasma insulin levels; consequently, HOMA-IR and HOMA-beta, which represent markers of insulin resistance and beta-cell function, respectively, display significant improvements after this dietetic intervention [71, 72]. An important benefit of VLCKD in improving insulin resistance is evident in youth obesity in particular [73, 74].

Goday *et al*. showed that as part of a weight loss program that included lifestyle and behavioral changes, a 12-week VLCKD resulted in higher adherence and satisfaction compared to a low-calorie diet [75]. Furthermore, a 12 months and parallel-group randomized (1:1) trial, performed in adults with prediabetes or type 2 diabetes, showed that an ad libitum very low-carbohydrate ketogenic diet was able to improve glycaemic control and reduce the dose and the number of drugs in comparison to a moderate carbohydrate, calorie-restricted low-fat diet [76].

Another and very original study by La Vignera *et al*. explored the effects of a 12-week VLCKD on body weight, indices of β-cell dysfunction, insulin resistance, and serum levels of Total Testosterone (TT) in overweight or obese male patients with metabolic hypogonadism, showing that VLCKD could be used to improve β-cell secretory function and insulin sensitivity [77].

Overweight and obesity lead the ß cells of the pancreas to a secretory effort, as they are called to release a greater amount of insulin to maintain glucose levels within the physiological range. Accordingly, hyperinsulinemia is present in the majority of patients with obesity [78]. Overweight/obesity causes the development of insulin resistance in peripheral tissues, which, in turn, increases even more the release of insulin. The secretory effort of the β-cells is responsible for the onset of a progressive functional failure that ultimately results in T2DM. In the Caucasian population, markers of β-cell dysfunction have been proposed as more reliable predictors of T2DM development than markers of insulin resistance [79]. Proinsulin, an insulin precursor with glucose-lowering effects, is one of the proposed markers. Proinsulin is increasingly released by pancreatic β-cells when they reach a late stage of deterioration [80, 81] as a further glucose-lowering attempt to delay the onset of T2DM. Large population studies with a duration of up to 27 years have reported increased serum proinsulin levels as predictors of T2DM development within 2-7 years [79, 82-84]. A disproportionate secretion of proinsulin has been described in patients with T2DM and has been implied as a strong predictor of β-cell dysfunction and insulin resistance [85]. A recent study has investigated for the first time the effects of VLCKD on proinsulin and proinsulin/insulin ratio. All patients enrolled in the study showed high proinsulin levels, which could reveal the presence of β-cell dysfunction and a predisposition to T2DM development. The authors of a study found VLCKD to be effective for body weight and insulin resistance and to induce normalization of proinsulin levels after 12 weeks of VLCKD-induced ketosis [86]. This suggests the safety of ketonemia for β-cells and the effectiveness of VLCKD in restoring β-cell dysfunction.

In a recent meta-analysis, the efficacy of a ketogenic diet for metabolic control was explored in patients with overweight or obesity with or without type 2 diabetes. This study confirmed ketogenic diet as more effective in improving metabolic parameters associated with glycaemic, weight, and lipid control in patients with overweight or obesity, especially those with pre-existing diabetes, as compared to low-fat diets [86].

Fig. (**3**), modified from a recent and exhaustive article [87], shows the theoretical mechanisms of KD's weight drop outcomes as follows: (1) appetite falls as a consequence of the effects of increased protein intake on appetite regulation hormones and direct appetite-suppressant action of ketone body; (2) lipogenesis reduction and lipolysis increase; (3) greater metabolic efficiency in the intake of fats signified by reduced resting respiratory quotient; (4) increased gluconeogenesis metabolic costs and protein thermal effects. Therefore, in light of the above, it can be confirmed that in patients with obesity and prediabetes, VLCKD reduces the glucotoxicity and insulin resistance phenomena. At the same time, it improves the functionality of the pancreatic β-cell and, consequently, glycometabolic control.

In conclusion, remission of prediabetes in patients with obesity is a possible event with non-pharmacological intervention using intensive weight management, such as VLCKD, for carefully selected patients, and it is more suitable if there is a need for rapid weight loss. The early intervention affects the success rates and VLCKD must always be performed under medical supervision [88].

## 5 TREATMENT OF OBESITY FOR THE PREVENTION OF DIABETES

Although lifestyle intervention is highly effective in preventing diabetes, it requires human (dietician, motor science graduate, psychologist) and economics resources rarely available in real life. Furthermore, adherence to lifestyle is difficult to maintain over time. For these reasons, diabetes prevention studies with pharmacological interventions using drugs for the treatment of obesity have been carried out. A summary of the effects of anti-obesity drugs on the risk of developing type 2 diabetes in patients with obesity and prediabetes is shown in Table **1**.

### 5.1 Orlistat

The drug selectively interacts with the lipases of the gastrointestinal tract, preventing their action. The inhibition of the enzyme lipase prevents the digestion of dietary lipids, creating a situation of malabsorption. The drug has poor systemic absorption and, therefore, its action is substantially limited to the gastrointestinal tract. The drug's role in diabetes prevention was evaluated in the XENDOS study. The prospective and multicentre study involved 3,304 subjects with normal glucose tolerance (79%) and with IGT (21%). Patients were randomly assigned to placebo or active treatment with orlistat 120 mg three times a day during a 4-year period of follow-up. All patients received lifestyle indications about reduction of the daily caloric intake and increase of physical activity. The primary endpoints of the study were the incidence of diabetes and changes in body weight. Secondary endpoints were changes in glycaemic parameters, waist circumference, plasma lipids, and blood pressure. The cumulative incidence of type 2 diabetes was 6.2% for the orlistat group and 9% for the placebo group, with a relative risk reduction of 37%.

The weight loss was - 6.9 kg for the orlistat group and -4.1 kg for the placebo group (*p* < 0.001 *vs*. placebo) and the reduction in mean abdominal circumference was 9.6 cm (after 1 year) and 6.4 cm (after 4 years) *versus* 7.0 and 4.4 cm, respectively, in the placebo group (*p* < 0.001 *vs*. placebo). The safety profile in the orlistat group was comparable to the placebo group except, as predictable for the mechanism of action of the molecule, for gastrointestinal disorders (91 *vs*. 65% of placebo). The latter, however, attenuated over the 4-year follow-up [89].

### 5.2 Liraglutide 3 mg

Liraglutide is an analogue of GLP 1, already used for the treatment of type 2 diabetes mellitus at the maximum dose of 1.8 mg. In parallel with glycaemic compensation, it induces a significant dose-dependent reduction in weight acting both centrally on the sense of satiety and at the gastric level by slowing down emptying. Liraglutide at a dose of 3 mg is approved for the treatment of obesity. The role of liraglutide in diabetes prevention was evaluated in the SCALE study. This study was a randomized, double-blind, placebo-controlled study involving adults with pre-diabetes and a body mass index of at least 30 kg/m^2^, or at least 27 kg/m^2^ in association with comorbidities. The study participants were randomized in a ratio of 2:1 to treatment with liraglutide 3.0 mg once daily subcutaneously or with placebo, in addition to treatment with a low-calorie diet and physical activity. The primary outcome was the time to onset of diabetes within 160 weeks of the study. By week 160, 26 (2%) of the 1472 individuals in the liraglutide group *versus* 46 (6%) of the 738 in the placebo group were diagnosed with diabetes while on treatment. The mean time elapsed from randomization to diagnosis was 99 (SD 47) weeks for the 26 in the liraglutide group *versus* 87 (SD 47) weeks for the 46 individuals in the placebo group. The time to onset of diabetes over 160 weeks among all randomized individuals was 2.7 times longer with liraglutide than with placebo (95% CI 1.9-3.9, *p* < 0.0001), corresponding to a hazard ratio of 0.21 (95% CI 0.13-0.34). The risk of diabetes was reduced by 66% compared to placebo treatment. The data is particularly relevant if we consider that for both groups, interventions were also made by promoting a better lifestyle through diet and physical activity. This aspect was confirmed by the weight loss (about -2% on average) obtained in the placebo group which, albeit limited, was in line with the literature data on the non-pharmacological treatment of long-term obesity. The SCALE study demonstrated that, out of every 3 prediabetic patients treated with liraglutide, one returned to normal glycaemia [90].

### 5.3 Semaglutide 2,4 mg

Semaglutide injection is an analogue of GLP 1, already used for the treatment of type 2 diabetes mellitus at the maximum dose of 1.0 mg. The role of semaglutide in diabetes prevention was evaluated in the STEP 1 study. STEP 1 study was a double-blind trial. The researchers enrolled 1961 adults with a body mass index of 30 or greater (≥27 in persons with ≥1 weight-related coexisting condition), who did not have diabetes. The study participants were randomly assigned, in a 2:1 ratio, to 68 weeks of treatment with once-weekly subcutaneous semaglutide (at a dose of 2.4 mg) or placebo, plus lifestyle intervention. Among participants with prediabetes at baseline, semaglutide was associated with improvements in glycated haemoglobin levels at week 68. 84.1% of participants in the semaglutide group with prediabetes at baseline, as compared to 47.8% in the placebo group, reverted to normoglycaemia [91].

### 5.4 Tirzepatide

In a post-hoc analysis of SURMOUNT-1 study, the 10-year predicted risk of developing T2D was calculated at baseline, week 24, and week 72 among participants randomized to receive 5, 10, or 15 mg tirzepatide or placebo. Tirzepatide treatment significantly reduced the 10-year predicted risk of developing T2DM (up to 69% at week 72) compared to placebo in participants with obesity or overweight, regardless of baseline glycaemic status particularly in patients with prediabetes [92].

### 5.5 Naltrexone/Bupropion

The two components of the drug act at the level of the arcuate nucleus of the hypothalamus and the mesolimbic dopaminergic gratification system. Bupropion is a weak inhibitor of neuronal dopamine and norepinephrine reuptake. It stimulates the hypothalamic neurons, producing Pro-opiomelanocortin (POMC), inducing an increase in energy expenditure and a reduction in the introduction of food. Naltrexone is an antagonist of μ-opiate receptors and acts by blocking the negative feedback established by β-endorphin, thus resulting in a more prolonged stimulation of POMC1 neurons. There is no data on diabetes prevention in studies conducted with this drug.

### 5.6 Bariatric Surgery

The role of bariatric surgery in the prevention of diabetes in severely obese subjects was evaluated in the Swedish Obese Subjects (SOS), a prospective study on the effects of surgically induced weight loss on the onset of diabetes, cardiovascular disease, and cancer. The SOS study involved 2010 subjects undergoing bariatric surgery and 2037 comparable for clinical characteristics undergoing medical therapy. The risk of developing diabetes in the surgically treated group compared to the conventional therapy group was reduced by 96%, 84%, and 78% at 2, 10, and 15 years of follow-up, respectively. The preventive effect on the onset of diabetes appears independent of the BMI at baseline; however, it was greater in patients with impaired fasting glucose in the initial evaluation compared to normoglycaemic subjects. The number of patients in need to undergo bariatric surgery to prevent one case of diabetes in the next 10 years was 1.3 in subjects with prediabetes and 7 in subjects with normal baseline glycaemia. Bariatric surgery can, therefore, represent a therapeutic choice for the prevention of diabetes in severe obesity [93].

## 6 USE OF GLUCOSE-LOWERING DRUGS TO PREVENT DIABETES AND TREAT OBESITY

### 6.1 Metformin

Metformin is a pharmacologic agent used for the treatment of patients with type 2 diabetes. It improves insulin action in the liver and skeletal muscle, therefore reducing blood glucose levels, with very low hypoglycaemic risk [94, 95]. It is generally well tolerated (the most common side effects are nausea and diarrhoea, but they usually disappear over time). A more serious but rare side effect is lactic acidosis, which can occur in patients with renal or liver insufficiency, congestive heart failure, or respiratory failure.

There is strong evidence that metformin is effective in preventing type 2 diabetes in patients with obesity with prediabetes [96]. The most convincing data were obtained from the Diabetes Prevention Program, a randomised controlled trial that enrolled 3234 subjects (BMI⌑ 24 kg/m^2^) at high risk of developing diabetes [37, 38]. The participants were assigned to an intensive lifestyle intervention, metformin plus standard lifestyle, or placebo plus standard lifestyle. Metformin dose was 850 mg twice daily, based on tolerability. Metformin was effective in reducing body weight (mean weight loss -2.1 kg for metformin *vs*. placebo). Patients in the metformin group had a lower risk of developing type 2 diabetes (relative risk reduction of -31% in the metformin group *vs*. placebo). The median delay in diabetes onset was 3 years in the metformin group [97].

Most patients enrolled in the DPP (88%) were included in the DPPOS, which evaluated long-term microvascular and macrovascular endpoints, as well as the risk of developing type 2 diabetes [38]. All patients received lifestyle counselling, and the placebo was stopped and metformin was continued in the group initially randomised to metformin in the DPP trial. Diabetes incidence rates (per 100 person per year) were 4.95 for metformin, and 5.6 for placebo. After 15 years of follow-up (considering DPP and DPPOS combined), metformin treatment showed a risk reduction of 18% of developing diabetes *vs*. placebo. Furthermore, a 28% reduction in microvascular complications was observed in all groups. It is important to note that metformin was more effective in diabetes risk reduction in the subgroup of patients with obesitywith a BMI⌑ of 35 kg/m^2^ and in younger patients.

Another study evaluated the effect of combination therapy with low-dose rosiglitazone and metformin [98] on diabetes risk prevention. The study enrolled 207 patients with impaired glucose tolerance randomly assigned to receive rosiglitazone (2 mg) and metformin (500 mg) twice daily or placebo for a median of 3.9 years. New onset diabetes was less frequent in the active treatment group than in the placebo group (14% *vs*. 39%; *p* < 0.0001). The relative risk reduction was 66% and the absolute risk reduction was 26% as compared to placebo.

### 6.2 GLP1 Receptor Agonists

GLP1 receptor agonists are glucose-lowering drugs that decrease HbA1c level, body weight, and blood pressure. They reduce appetite and slow gastric emptying, thereby promoting significant weight loss. They are associated with gastrointestinal side effects, usually transitory and dose-related (nausea, vomiting, diarrhea), and low hypoglycaemic risk. The beneficial effect of GLP1 receptor agonists on body weight can be of particular interest in patients with obesity. The dosages used in the treatment of obesity are higher than those used for the treatment of diabetes. Diabetes prevention studies have used dosages for the treatment of obesity and, therefore, have already been addressed. The only study with dosages used for diabetes treatment is for exenatide LAR [99]. This study on obese non-diabetic patients was conducted with exenatide LAR. It was a 24-week trial that included 152 obese participants at high risk of developing diabetes, randomised to exenatide LAR 2 mg weekly or placebo. Exenatide group lost more body weight and normalized hyperglycaemia more frequently as compared to the placebo (77% *vs*. 56%, respectively) [99].

New interesting data in the near future will be received from a new class of incretin compounds, the dual GLP1-GIP agonists. Tirzepatide, which is one drug of the class, has proven to be even more effective in body weight reduction in type 2 diabetes patients as compared to semaglutide [100]. It is sold in the US and it should soon be available also in Europe.

### 6.3 SGLT2 Inhibitors

SGLT2 inhibitors are new pharmacological agents used for the treatment of type 2 diabetes. They reduce hyperglycaemia by increasing renal glucose excretion and inducing glycosuria [101]. They are effective in reducing body weight and blood pressure.

Following several data from RCTs, SGLT2 inhibitors showed significant renal and cardiovascular benefits, regardless of the presence of diabetes. The beneficial effects of SGLT2 inhibitors on body weight and glycaemic control can be of interest in patients with obesity with prediabetes, but, so far, there is limited data in this clinical context.

The first study assessed the effects of combination therapy with exenatide LAR 2 mg weekly and dapagliflozin 10 mg daily in patients with obesity without diabetes. This pilot study, with a short follow-up (24 weeks), revealed greater weight loss and less frequency of prediabetes in the group treated with exenatide/dapagliflozin as compared to placebo [102]. The DAPA-HF (Dapagliflozin and Prevention of Adverse outcomes in Heart Failure) is an RCT that evaluated the cardiovascular benefits of dapagliflozin in patients with heart failure [103]. An exploratory analysis of the DAPA HF assessed the effects of dapagliflozin on diabetes risk in the subgroup of overweight patients without diabetes at study entry [104]. After a median follow-up of 18 months, diabetes occurred in 7.1% of the placebo and 4.9% of the dapagliflozin group, corresponding to a 32% reduction in diabetes incidence with dapagliflozin.

This result was not observed in the Emperor study with empagliflozin. In this randomised trial, patients with heart failure were treated with empagliflozin for a median of 26.2 months. In the prediabetes subgroup, the occurrence of new-onset diabetes was similar as compared to placebo [105]. However, it should be noted that the incidence of type 2 diabetes was not a pre-specified end point of the study.

A pooled analysis of data from DAPA-CKD and DAPA-HF [106] has recently been published to assess the effect of dapagliflozin on diabetes risk. DAPA-CKD is an RCT that evaluated the renal benefits of dapagliflozin in patients with chronic renal disease, independently of the presence of diabetes [107]. The pooled analysis included 4003 participants (1398 from DAPA-CKD and 2605 from DAPA-HF) with no previous history of diabetes, followed up for 21 months [106]. Diabetes developed less frequently in the dapagliflozin group as compared to the placebo group (4.35 and 6.3% of patients, respectively).

### 6.4 Pioglitazone

Pioglitazone is an insulin-sensitizing drug that ameliorates glycaemic control in type 2 diabetes, reduces triglycerides, and increases HDL levels, with low hypoglycaemic risk. However, it can induce body weight gain and water retention [108].

The ACT NOW (Advancing Clinical Trials in Neonatal Opioid Withdrawal) study evaluated the effect of pioglitazone on type 2 diabetes prevention [109]. In this trial, 602 subjects with prediabetes (BMI >25 kg/m^2^) were randomised to pioglitazone 45 mg/day or placebo and were followed up for 24 months. Treatment with pioglitazone normalized glucose levels in 48% of patients as compared to 28% in the placebo group. Diabetes developed in 45 of 228 individuals (19.7%) in the placebo group and 15 of 213 individuals (7.0%) in the PGZ group during a median follow-up period of 2.4 years. The improvement of glycaemic status was associated with increase in insulin sensitivity and beta-cell function [110]. Another study evaluated the diabetes risk after discontinuation of pioglitazone in the population of the ACT NOW trial [111]. After cessation of therapy, the protective effect of pioglitazone reduced, since new onset diabetes occurred in similar percentage of individuals in placebo and pioglitazone group (12.3% and 11.2%, respectively).

### 6.5 Acarbose

Acarbose is an alpha-glucosidase inhibitor that can be used in the treatment of diabetes, and is effective in lowering post-prandial hyperglycaemia. It is associated with frequent side effects, primarily flatulence and diarrhea. Data from clinical trials have proven that acarbose can delay the occurrence of diabetes in high-risk individuals [112-114].

The STOP-NIDDM was a multicentre, placebo-controlled trial that included 1368 patients with impaired glucose tolerance (mean BMI 25.4 kg/m^2^), randomised to acarbose 100 mg three times daily or placebo [112]. Diagnosis of new-onset diabetes was less frequent in the acarbose group as compared to the placebo group (32% *vs*. 42% of patients, respectively). More patients treated with acarbose normalized hyperglycaemia as compared to the placebo group.

The beneficial effects of acarbose on diabetes risk were confirmed in the ACE trial (Acarbose Cardiovascular Evaluation), which was a randomised study on Chinese people with coronary heart disease and impaired glucose tolerance [113]. A total of 6522 patients were allocated to acarbose 50 mg or placebo. At the end of the study, acarbose-treated patients had an 18% relative risk reduction in diabetes onset.

### 6.6 Additional Considerations

International guidelines are recently paying more attention to implementing strategies for type 2 diabetes prevention in high-risk populations. AACE stated that “the primary goal of prediabetes management is weight loss in patients with overweight or obesity” [115]. Metformin, alfa-glucosidase inhibitors, thiazolidinediones, GLP1-receptor agonists, and SGLT2 inhibitors have proven efficacy in reducing the incidence of type 2 diabetes in subjects with prediabetes. For some of these drugs, we need more data about cost-effectiveness and long-term efficacy in type 2 diabetes prevention.

Two GLP1 receptor agonists, liraglutide and recently semaglutide, have been approved in the United States and Europe for the treatment of obese (BMI ≥30 kg/m^2^) or overweight (BMI ≥27 kg/m^2^) patients with comorbidities (high blood pressure, dyslipidemia, type 2 diabetes). So far, no medication has an indication for diabetes prevention in the United States or Europe. Nevertheless, international scientific societies highlight that pharmacotherapy can be effective in reducing progression to diabetes in obese individuals with prediabetes [115], in addition to the implementation of lifestyle strategies. ADA recommends that metformin use for the prevention of type 2 diabetes should be considered in patients with prediabetes, especially for those with BMI >35 kg/m^2^, age lower than 60 years, fasting glucose >100 mg/dl (6 mmol/L) or HbA1 >6.0% (>42 mmol/mol), and women with prior gestational diabetes mellitus [8]. A summary of the effects of glucose-lowering drugs on the risk of developing type 2 diabetes in patients with obesity and prediabetes is shown in Table **2**.

## 7 TREATMENT OF HYPERTENSION AND BLOOD PRESSURE IN PATIENTS WITH PREDIABETES

Prospective studies have documented a potential reciprocal relationship between hypertension and alterations in glucose metabolism. Nondiabetic hypertensive patients have a high prevalence of prediabetes [116]. A meta-analysis of 30 prospective studies suggested that each 20 mm Hg increment of systolic blood pressure increases the risk of new-onset type 2 diabetes by 77% [117]. A longitudinal population-based study conducted in Shangai, China, between 2004 and 2014, investigated if pre-diabetes (defined by the authors as IGR-impaired glucose regulation) alone or combined with hypertension could promote cardiovascular disease. Isolated IGR was found to be non-associated with elevated cardiovascular risk. IGR and hypertension in the same individual significantly increased the risk of developing both diabetes and cardiovascular disease with an adjusted OR of 2.41 for cardiovascular disease and 6.37 for diabetes mellitus. The authors concluded that glycaemic values in the prediabetes range lead to a significant increase in cardiovascular risk when associated with other risk factors, especially hypertension [118]. A meta-analysis of 13 randomized clinical trials enrolling 37,736 subjects with diabetes or impaired glucose tolerance showed intensive SBP control (target < 135 mmHg) as associated with a 10% reduction in all-cause mortality and a 17% reduction in stroke, with similar outcomes for other macrovascular and microvascular (cardiac, renal, and retinal) events compared to standard SBP control (target < 140 mmHg). More intensive BP control (≤ 130 mmHg) was associated with a greater reduction in stroke, but it did not reduce other events [119].

The guidelines recommend blood pressure targets that are not different between subjects with diabetes or prediabetes. The ESC guidelines indicate a target for systolic blood pressure of 130 mmHg or < 130 mmHg if well tolerated and a target for diastolic blood pressure <80 mmHg. A target of < 120/70 is not recommended [120].

Particular focus is suggested on the choice of the hypotensive drug in prediabetes. In prediabetes, the risk of new-onset diabetes is lower with RAAS blockers than with beta-blockers or diuretics [120]. The ADAPT study is an open, prospective parallel-group study comparing patients treated with an ACE inhibitor *versus* a diuretic-based treatment. The principal aim was to document the first manifestation of type-2 diabetes in either group. New-onset diabetes was less frequent in the ramipril than in the diuretic group over 4 years. Differences were statistically different at a median duration of 3 years (24.4% *vs*. 29.5%; *p* < 0.05). The authors concluded that ramipril treatment is preferable over diuretic-based treatment regimens for the treatment of hypertension in pre-diabetic patients because new-onset diabetes is delayed [121]. This finding was confirmed in a recent meta-analysis, in which the data were pooled to investigate the differential effects of five major classes of antihypertensive drugs on the risk of new-onset type 2 diabetes. Overall, data from 22 studies conducted between 1973 and 2008 were obtained by the Blood Pressure Lowering Treatment Trialists' Collaboration (Oxford University, Oxford, UK). Systolic blood pressure reduction by 5 mmHg decreased the risk of type 2 diabetes by 11% (hazard ratio 0.89 (95% CI 0.84-0.95)). The evaluation of effects of five major classes of antihypertensive drugs showed that in comparison to placebo, angiotensin-converting enzyme inhibitors (RR 0.84 (95% 0.76-0.93)) and angiotensin II receptor blockers (RR 0.84 (0.76-0.92)) reduced the risk of new-onset type 2 diabetes; the use of β blockers (RR 1.48 (1.27-1.72)) and thiazide diuretics (RR 1·20 (1.07-1.35)) increased this risk, and no effect was found for calcium channel blockers (RR 1.02 (0.92-1.13)) [122].

In conclusion, subjects with prediabetes have the same blood pressure targets as diabetic subjects. These objectives should preferably be achieved with the use of RAAS inhibitors, which have shown a favorable effect on the prevention of type 2 diabetes.

## 8 TREATMENT OF DYSLIPIDAEMIA AND LIPID TARGETS IN PATIENTS WITH PREDIABETES

Dyslipidaemia in obesity and prediabetes is characterized by reduced HDL-C and elevated fasting Triglyceride (TG) concentrations; however, increased Low-density Lipoprotein Cholesterol (LDL-C) and normal to marginally increased total cholesterol are not uncommon among these individuals. The effects of obesity on lipid metabolism are dependent on the location of the adipose tissue [123]. Increased visceral adipose tissue is associated with higher levels of triglycerides and lower levels of HDL-C. Moreover, increased visceral adipose tissue is associated with insulin resistance, which may contribute to the lipid changes described hereafter.

Lipid abnormalities commonly seen in patients with obesity and prediabetes encompass elevated levels of triglycerides, VLDL, Apo B, and non-HDL-cholesterol, in contrast to low levels of HDL-cholesterol and Apo A-I. LDL-C levels are usually normal, but small dense LDL (sdLDL) particles are often increased in these patients [123]. These sdLDL particles are pro-atherogenic since they have a decreased affinity for the LDL receptor resulting in a prolonged period of time in the circulation. Moreover, sdLDL particles could enter the arterial wall and are more susceptible to oxidation, which could result in an enhanced uptake by macrophages. Postprandial triglyceride levels are also increased in obese subjects with prediabetes and these chylomicron remnants are pro-atherogenic [123]. The increased risk for cardiovascular disease in patients with obesity and prediabetes is partially accounted for by this dyslipidaemia.

Consequently, the purpose of treating lipid disorders in patients with obesity and prediabetes is to prevent the development of cardiovascular disease and other diseases. A number of guidelines have been published that discuss in detail cardiovascular risk assessment and provide recommendations on treatment strategies [124-128]. It should be noted that while these guidelines are similar, there are significant differences between their recommendations. Noteworthily, it should be pointed out that no specific guidelines address dyslipidemia specifically in obesity and prediabetes. The current American College of Cardiology/American Heart Association (ACC/AHA) guidelines do not recommend specific lipid targets but rather treatment with statins to lower LDL-C by a certain percentage [125]. In contrast, other guidelines, such as the National Lipid Association, International Atherosclerosis Society, European Society of Cardiology/European Atherosclerosis Society, and American Association of Clinical Endocrinologists (AACE), do recommend specific LDL-C and non-HDL-C goals depending upon the cardiovascular risk assessment [124, 126-129]. In addition to reducing CV risk in these patients, the treatment of hypertriglyceridemia is aimed at lowering the risk of pancreatitis [123]. The National Lipid Association recommends treating triglyceride levels greater than 500 mg/dl while the Endocrine Society recommends treating triglycerides if they are greater than 1000 mg/dl to lower the risk of pancreatitis [128, 130].

Most of the current guidelines are concordant in recommending to achieve LDL-C levels less than 70 mg/dl and non-HDL-C levels less than 100 mg/dl in patients with cardiovascular disease or patients at very high risk for the development of cardiovascular disease. Furthermore, AACE and the European Society of Cardiology/European Atherosclerosis Society have recommended LDL-C levels less than 55 mg/dl in patients at extreme or very high risk [128, 129]. In other patients, an LDL-C level less than 100 mg/dl and a non-HDL-C level less than 130 mg/dl is a reasonable goal. Obese prediabetic patients could be included in this latter reasonable goal. Indeed, patients with obesity and/or prediabetes are at a lower risk than those with overt diabetes, so one could tolerate slightly higher LDL levels; a target LDL of less than 100 mg/dl is probably appropriate for this group. Accordingly, a reasonable proposal could be to recommend that these individuals be followed regularly and assessed for lipid treatment in the same way as would their euglycaemic and/or normal-weight counterparts.

Lastly, even if most current guidelines stress the importance of LDL as the primary target of treatment, TG levels should also be considered to reduce the residual CVD risk of these patients. Indeed, CVD risk is increased when fasting TGs are >1.7 mmol/L (>150 mg/dL), but the use of drugs to lower TG levels should only be considered in high-risk patients when TGs are >2.3 mmol/L (>200 mg/dL) [127].

### 8.1 Statins and Risk of Diabetes

In February 2012, the US Food Drug Administration (FDA) issued a warning related to statin therapy and an increased risk of new-onset diabetes [131]. At that time, most of the evidence emerged from post-hoc analyses of studies not specifically designed to investigate the relationship between statin and the risk of diabetes.

In 2017, a retrospective study included 308 subjects (265 on statins and 43 controls on diet) with a follow-up of 5-15 years. It investigated the risk of altered glucose levels and New-onset Diabetes (NOD) associated with statins according to glucose levels at baseline in a population treated for dyslipidemia on primary prevention for >5 years. The cumulative incidence of NOD was 13.6% (9.3% in controls and 13.5% in treated patients). NOD was diagnosed after 3.4±1.8 years. This study confirmed that statins are associated with an increased risk of NOD or altered glucose levels, mainly in subjects with fasting glucose >100 mg/dl before initiating the treatment. The metabolic phenotype, lifestyle risk factors, and family history of type 2 diabetes have been reported to contribute to the risk [132].

Recent data from 14 randomized control trials meta-analysis have suggested a 9,33% higher risk of type 2 diabetes with statin use. Also, this study has shown that that patient populations with a greater predisposition to diabetes mellitus and those with thicker epicardial adiposity are more at risk for the development of statin-induced diabetes. Despite these observations, robust data from a variety of investigations suggest that the CVD-preventative benefits of statin treatment significantly outweigh the risks associated with the development of diabetes [133].

The diabetogenic effect is common to all statins except for pitavastatin. Meta-analyses in which patients were stratified by statin type (atorvastatin, lovastatin, pravastatin, rosuvastatin, simvastatin) found no differences in the incidence of diabetes between the various molecules [134, 135]. There could instead be a dose-dependent effect. A meta-analysis investigated the risk of diabetes by comparing intensive-dose (simvastatin 80 mg, atorvastatin 80 mg) or moderate-dose statin therapy (simvastatin 20-40 mg, atorvastatin 10 mg, pravastatin 40 mg). In 5 statin trials with 32752 participants without diabetes at baseline, 2749 developed diabetes (1449 assigned intensive-dose therapy and 1300 assigned moderate-dose therapy, representing 2.0 additional cases in the intensive-dose group per 1000 patient-years); as compared to moderate-dose statin therapy, the Number Needed to Harm (NNH) per year for intensive-dose statin therapy was 498 for new-onset diabetes while the number needed to treat per year for intensive-dose statin therapy was 155 for cardiovascular events [136]. Conversely, in a retrospective, multicenter active-comparator, new-user, cohort study, pitavastatin reduced the risk of type 2 diabetes compared to atorvastatin or rosuvastatin. Data from electronic health records were used to identify new users of pitavastatin, atorvastatin, or rosuvastatin (duration of therapy > 180 days) without a history of diabetes at baseline with HBA1c level < 5.7%. The meta-analysis of the HRs demonstrated that pitavastatin resulted in a significantly reduced risk of type 2 diabetes than atorvastatin + rosuvastatin (HR 0.72; 95% CI 0.59-0.87) especially compared to low-to-moderate intensity atorvastatin + rosuvastatin users (HR 0.78; CI 0.62-0.98) [137]. Regarding the possible explanations, some studies have shown a smaller reduction in insulin secretion in beta cells exposed to pitavastatin compared to atorvastatin and rosuvastatin. Furthermore, glucose uptake in skeletal muscle is greater with pitavastatin compared to atorvastatin and rosuvastatin. Pitavastatin also increases adiponectin levels, reducing insulin resistance [138-141].

These observations underline the importance of careful follow-up of patients with risk factors for diabetes to be treated with statins. In these patients, attention must be paid to the correct lifestyle to avoid weight gain, sedentary lifestyle, and modifiable risk factors of evolution towards diabetes. Where available, pitavastatin could represent the drug of first choice in patients at high risk of diabetes.

## 9 NON-ALCOHOLIC FATTY LIVER DISEASE (NAFLD) IN PREDIABETES

The terms NAFLD and Nonalcoholic Steatohepatitis (NASH) had long been considered unsatisfactory and this led to a consensus document on new fatty liver disease nomenclature in 2023 [142]. In this document, the term Steatotic Liver Disease (SLD) describes any hepatic steatosis identified by imaging or biopsy, irrespectively of the aetiology. SLD includes a series of subcategories [142].

When hepatic steatosis is detected in patients that present at least one cardiometabolic risk factor, in the absence of other recognizable cause, SLD is called Metabolic dysfunction Associated Steatotic Liver Disease (MASLD); if MASLD and steatohepatitis are present, the term steatohepatitis associated with metabolic dysfunction (MASH) will be used.

The cardiometabolic criteria for the definition of MASLD are as follows: Body Mass Index (BMI) ≥ 25 (23 for Asian populations); waist circumference > 94 cm in males and > 80 cm in females, or ethnicity adjusted equivalent; prediabetes or type 2 diabetes mellitus; blood pressure ≥ 130/85 mmHg or specific antihypertensive drug therapy; plasma triglycerides ≥ 1.70 mmol/l (150 mg/dl) or lipid-lowering therapy; plasma HDL-C ≤1.0 mmol/l (40 mg/dl) in males or ≤1.3 mmol/l (50 mg/dl) in females or lipid-lowering therapy.

In patients with hepatic steatosis in the absence of manifested cardiometabolic risk factors, tests may be useful to evaluate whether insulin resistance is present and thus identify those with possible MASLD.

In this review, to avoid misunderstandings, we have referred to the traditional nomenclature in use until 2023, and NAFLD, NAFL, and NASH have been used throughout the text.

**Fig. (4):**
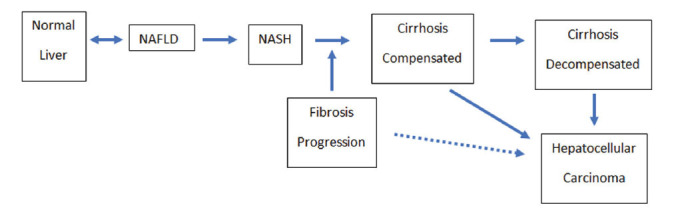
Spectrum of possible evolution of NAFLD.

NAFLD is defined by the presence of steatosis in more than 5% of hepatocytes in the absence of excessive alcohol intake (> 30 g per day for men and > 20 g per day for women) or chronic liver disease [143]. NAFLD encompasses a spectrum of diseases, including Non-alcoholic Fatty Liver or simple steatosis (NAFL), which has a more benign course, and Non-alcoholic Steatohepatitis (NASH), which can progress from fibrosis to cirrhosis to Hepatocellular Carcinoma (HCC) [144-147] (Fig. **4**). The most relevant and common risk factors for the development of NAFLD are metabolic syndrome, dyslipidaemia, obesity, diabetes, and prediabetes. The incidence and prevalence of NAFLD are rapidly rising worldwide and it is now the most common cause of chronic liver disease, the second leading cause of end-stage liver disease, and the second most common cause of primary liver cancer among adults waiting for liver transplantation [144]. The presence of prediabetes in people with NAFLD is higher compared to that of the general population [148]. In patients with prediabetes, NAFLD increases the risk of type 2 diabetes; in turn, obesity, type 2 diabetes, and NAFLD, when coexisting, act synergistically to increase the risk of adverse hepatic and extrahepatic outcomes [149].

### 9.1 NAFLD and Fibrosis According to Glucose Status

In a study on 2090 patients with normoglycaemia, prediabetes, or diabetes assessed by means of magnetic resonance elastography, the risk of liver fibrosis according to glucose status was observed. Compared to normoglycaemic patients, those with diabetes had a higher risk of significant fibrosis (adjusted Odds Ratio (aOR): 3.02, 95% Confidence Interval (CI): 1.57-5.81; *p* < 0.001). However, there was no difference between prediabetes and normoglycaemia (aOR: 1.05, 95% CI: 0.59-1.86; *p* = 0.876). A subgroup analysis also showed that prediabetes, unlike diabetes, was not associated with significant fibrosis in subjects with or without liver disease [150].

A recent study analysed the prevalence of NAFLD in 521 patients with severe obesity (mean BMI 51.87 kg/m^2^), undergoing bariatric surgery. Intraoperative liver biopsies were performed and histopathologic findings were correlated with their baseline glucose status. Of the 521 patients, 439 (84.3%) were women, with a mean age of 41.8 ± 10 years. Patients were classified into 3 groups according to their baseline glucose status: Group 1 (G1) was composed of 167 (32.05%) patients with type 2 diabetes, Group 2 (G2) included 132 (25.33%) patients with prediabetes, and Group 3 (G3) comprised 222 (42.61%) normoglycaemic patients. The results of the study showed that in patients with severe obesity, prediabetes status was observed in 25.3% of patients; in these patients, the prevalence of steatohepatitis was 49.2%, close to the prevalence observed in patients with diabetes (59.4%), and higher compared to that of normoglycaemic patients (36%) (*p* < 0.001). The presence of fibrosis, on the other hand, in patients with prediabetes, was only 29.2%, similar to that of normoglycaemic patients (28,6%) and much lower than patients with diabetes (56,4%) (*p* < 0.001) [151].

### 9.2 Diagnosis and Clinical Assessment

#### 9.2.1 NAFLD

NAFLD is most often diagnosed by imaging, although it can be inferred from clinical risk scores (*e.g*., fatty liver index) or identified histologically. In routine practice, the most used test is abdominal ultrasonography [144]. The ability to quantify steatosis by measuring ultrasonic attenuation of the echo wave, termed Controlled Attenuation Parameter (CAP), has been implemented on the FibroScan device. CAP is a promising point-of-care technique for rapid and standardized steatosis detection, with high applicability (>95%) when using the XL probe. MRI-based measurements of hepatic steatosis (*e.g*., MRI Proton Density Fat Fraction, MRI-PDFF) can detect as little as 5% fat and are sensitive to dynamic change [144, 152, 153]. MRI-PDFF is, therefore, the most accurate non-invasive method for detecting and quantifying steatosis, but it is not recommended as a first-line tool given its cost and limited availability, and it is more suited to clinical trials [154]. Clinicians must be aware that liver enzyme concentrations can be normal in more than half of patients with NAFLD, and correlate poorly with the histological severity [144, 155].

#### 9.2.2 Liver Fibrosis

The assessment of the presence and severity of hepatic fibrosis, which correlates with prognosis, is of particular importance [154]. Liver biopsy is still the reference standard, but it is invasive, and can lead, even if rarely, to severe complications; furthermore, it is prone to sampling bias, with intraobserver and interobserver variability in histological assessment and being relatively expensive [144, 156-158]. This has led to the development of several non-invasive tests for staging liver fibrosis [154, 159]. Fibrosis scores are based on demographic, clinical, and routine laboratory parameters. They are inexpensive to use and have moderate accuracy but high negative predictive values to exclude advanced liver fibrosis, especially in community and primary care settings [144, 152, 154, 160]. In this context, they can be useful to define patients with low fibrosis scores who are at low risk of developing liver-related complications [161]. The most validated are NAFLD Fibrosis Score (NFS) and Fibrosis-4 (FIB-4) [144, 159]. Automatic calculation and systematic reporting of simple non-invasive fibrosis tests, such as FIB-4, are now recommended in primary care [156] to rule out advanced fibrosis. To estimate liver fibrosis in NAFLD, the measure of liver stiffness by ultrasound-based elastography and magnetic resonance elastography can also be used [144, 156]. Magnetic Resonance Elastography (MRE), given its cost and limited availability, is more suited for use by specialists in tertiary referral centres and for research purposes [156].

### 9.3 Treatment

#### 9.3.1 Pioglitazone

Pioglitazone is a selective ligand of Peroxisome Proliferator-activated Receptor (PPAR)-gamma, expressed at a high level on adipose tissue and also on Kupffer cells, and possesses great anti-inflammatory properties. A recent systematic review concluded that pioglitazone can significantly alleviate NASH in patients with prediabetes, improving steatosis, inflammation, and ballooning grade. Pioglitazone can also significantly improve insulin resistance and reduce fasting blood glucose, glycosylated haemoglobin, plasma AST and ALT. Pioglitazone is known to have side effects and contraindications (such as heart failure) and physicians must be familiar with the use of pioglitazone in these patients. With respect to liver fibrosis, pioglitazone (45 mg/die for 18 months) proved effective only in one RCT on patients with prediabetes or diabetes [162, 163].

#### 9.3.2 Other Glucose-lowering Agents

A recent network meta-analysis assessed the efficacy of glucose-lowering agents for the treatment of NAFLD in patients with prediabetes or diabetes. The study concluded that thiazolidinediones (in particular, pioglitazone) are the most effective in normalizing liver metabolism, although they are associated with weight gain; GLP1 RAs are very promising in these patients and might be used in combination with pioglitazone to improve the clinical outcome and limit weight gain. Metformin has also been reported to improve liver metabolism although to a lower extent without inducing histological improvement [164].

Empagliflozin has been tested for the treatment of NAFLD in non-diabetic patients with promising results for the reduction of steatosis and fibrosis [165].

#### 9.3.3 Weight Loss and Lifestyle

Lifestyle changes, always at the basis of any intervention on weight and glycaemic status, have been proven effective in reducing Hepatic Fat Content (HFC) in patients with obesity and prediabetes with NAFLD. A study on 115 pre-diabetic patients aged 50-65 years demonstrated that Aerobic Exercise (AEx) training in combination with a fibre-enriched diet can reduce HFC and improve glycaemic control. HFC was significantly reduced in the AEx (-24.4%), diet (-23.2%), and aerobic exercise + diet (AED) (-47.9%) groups in contrast to the 20.9% increase in the control group (*p* = 0.001 for all); only AED group exhibited significantly decreased HbA1c level (-4.4%, *p* = 0.01) compared to the control group (-0.6%) [166].

### 9.4 Conclusive Remarks: Importance of Diagnosis MASLD in Prediabetes

In the past, there was no full agreement between the different scientific societies with respect to screening for NAFLD, partly reflecting the paucity of available effective therapeutic interventions and the existence of concerns about the possible consequences of overdiagnosis of NAFLD. More recently, interest in this pathology has been constantly and quickly growing [147, 167-169].

European Societies, together with others, support screening in high-risk populations [143, 170]. The American Diabetes Association’s 2023 Standards of Medical Care in Diabetes recommend evaluating patients with prediabetes, as well as T2D, with steatosis or elevated ALT, for NASH and fibrosis [171]. A more recent clinical practice guideline of the American Association of Clinical Endocrinology (AACE) co-sponsored by the American Association for the Study of Liver Diseases (AASLD) recommends that clinicians should consider people with prediabetes to be “high risk” and screen them for NAFLD and advanced fibrosis [169]. On the other hand, patients with obesity and NAFLD must be screened for prediabetes.

In patients with prediabetes, the prevalence of fibrosis is low and the progression of NAFLD often follows the development of overt diabetes. It is, therefore, important to identify patients with obesity, NAFLD, and prediabetes because effective treatment of prediabetes and NAFLD can be undertaken for possible prevention of fibrosis. Probably, the most effective approach can be the use of pioglitazone for the treatment of NAFLD, in combination with liraglutide/semaglutide for diabetes prevention, providing an additional benefit for the treatment of NAFLD and the prevention of weight gain due to the use of pioglitazone.

## CONCLUSION

Obesity and prediabetes affect a consistent portion of the general population, but are largely underdiagnosed, underestimated, and undertreated. Macrovascular complications in patients with diabetes are mainly caused by insulin resistance. The difference between prediabetes and diabetes is the decline in residual beta-cell function, not insulin resistance that starts several years before the onset of diabetes. This is why in prediabetes, the occurrence of macrovascular complications and the overall cardiovascular risk is by all means similar to that of patients with diabetes. This is why, it is important to identify prediabetes and treat patients not only to prevent or delay the onset of diabetes, but to reduce the cardiovascular risk associated with prediabetes.

Management of these patients must focus on the following:

- Body weight control: This is the single most relevant intervention that leads to the normalisation of glycaemic status and prevention of complications and diabetes. Lifestyle interventions (eating habits and physical activity) remain indispensable but often not enough for substantial and prolonged weight loss and risk reduction; physicians must be confident of the prescription of drugs designed for weight control and, when appropriate, of bariatric/metabolic surgery.
- Reduction of insulin resistance and glycaemic values: Adding insulin-sensitizing drugs or other glucose-lowering drugs with an effect on weight control must be considered.
- Normalization of lipids and blood pressure: The addition of specific drugs to treat according to the level of risk employing the same targets used for diabetes must be taken into account.
- Early recognition of MASLD: This should be done for early treatment and prevention of fibrosis.

Too often these days, physicians are not aware of the impact of obesity and prediabetes on their patients and only offer follow-up to detect the onset of diabetes when it might be too late to prevent macrovascular complications. Therefore, it is necessary to raise awareness of the clinical relevance of this pathological condition in order to prompt early intervention before complications occur.

## Figures and Tables

**Fig. (3):**
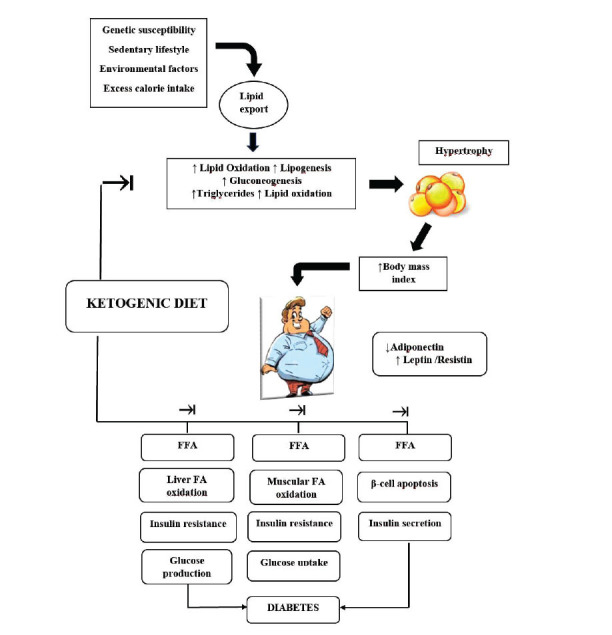
Ketogenic diet targets pathological mechanisms linking obesity and diabetes mellitus.

**Table 1:** Effect of anti-obesity drugs/surgery/lifestyle on the prevention of type 2 diabetes in patients with obesity and prediabetes.

**Drug/Surgery/Lifestyle**	**Diabetes Prevention/Delay**	**Effect**	**Study/References**
Orlistat	Yes	RRR 37% in 4 yrs	Xendos/89
Liraglutide 3 mg	Yes	RRR 66% in 3 yrs	Scale/90
Semaglutide 2,4 mg	Yes	84.1% of treated patients normoglycemic after 68 wks (*vs*. 47.8% in controls)	Step 1/91
Tirzepatide 15 mg	Yes	69% reduction (at 72 weeks) of the 10 yrs. predicted risk of developing T2DM	Post hoc analysis Surmount 1/92
Naltrexone/bupropione	Not known	Not known	No study available
Bariatric surgery	Yes	RRR 96%, 84% and 78% at 2, 10 and 15 yrs.	SOS/92
Lifestyle	Yes	RRR 42% in 6 yrs.	Da Qing DPOS/59
Lifestyle	Yes	RRR 58/34% in 3/10 yrs.	DPP/9-60
Lifestyle	Yes	RRR 58% in 4 yrs.	Finnish DPP/32

**Abbreviations:** RRR: relative risk reduction; DPP diabetes prevention program; DPOS diabetes prevention outcome study.

**Table 2:** Effect of glucose-lowering drugs on the prevention of type 2 diabetes in patients with obesity and prediabetes.

**Drug**	**Diabetes Prevention/Delay**	**Effect**	**Study/References**
Metformin	Yes	RRR 31/18% in 3/10 yrs	DPP/9-60
Pioglitazone	Yes	RRR 64% in 2,4 yrs	ACT NOW/110
Dapaglifozin	Yes	RRR 31% in 21 mts	Pooled analysis of DAPA HF-DAPA CKD/106
Empaglifozin	No	No	Emperor/105
Acarbose	Yes	RRR 23.8% in 3.9 yrs	STOP-NIDDM/112

**Abbreviations:** RRR: relative risk reduction; DPP diabetes prevention program.

## LIST OF ABBREVIATIONS

ARIC: Atherosclerosis Risk in Communities
DPP: Diabetes Prevention Program
IS: Insulin Sensitivity
MASLD: Metabolic Dysfunction-associated Steatotic Liver Disease
SFA: Saturated Fatty Acids
T2DM: Type 2 Diabetes Mellitus
